# Opportunities in stroke care at safety net hospitals: A socioecological perspective

**DOI:** 10.1016/j.neuros.2025.100005

**Published:** 2025-06-04

**Authors:** Anjail Sharrief, Joshua Wollen, Maha Almohamad, M. Carter Denny, Erica Jones, Aardhra M. Venkatachalam, Digvijaya Navalkele, Shivika Chandra, Mariam Ahmed, Robert Pratt, Bradley D. Shy, John McMenamy, Chigozirim Izeogu, Lesli E. Skolarus, Nneka L. Ifejika, Nicole R. Gonzales

**Affiliations:** a Department of Neurology, McGovern Medical School at The University of Texas Health Science Center at Houston (UTHealth); b University of Houston College of Pharmacy, Department of Pharmacy Practice and Translational Research; c Department of Neurology, McGovern Medical School at The University of Texas Health Science Center at Houston (UTHealth), Center for Health Equity UTHealth; d Department of Neurology, Georgetown University Medical Center and MedStar Health: Washington, D.C; e University of Texas Southwestern Medical Center, Department of Neurology; f Queen’s University College of Medicine, Kennesaw GA; g Emory University School of Medicine, Department of Neurology; h Smith College, Northampton, MA; i Department of Neurology, University of Colorado School of Medicine, Aurora, CO; j Department of Emergency Medicine, Denver Health Medical Center, University of Colorado School of Medicine, Aurora, CO; k Department of Radiology, Denver Health, Denver, CO; l The Ken & Ruth Davee Department of Neurology, Northwestern University Feinberg School of Medicine, Chicago, IL; m Division of Academics, Ochsner Health System, New Orleans, LA; n Department of Physical Medicine and Rehabilitation, University of Texas Southwestern Medical Center, Dallas, TX; o Department of Neurology, Neurohospitalist & Stroke Section, University of Colorado School of Medicine, Aurora, CO, United States of America

**Keywords:** Safety-net hospital, Stroke disparities, Stroke inequities, Public hospitals

## Abstract

Safety net hospitals (SNHs) provide care to patients regardless of their insurance status or ability to pay, serving populations at the highest risk for poor stroke outcomes. These include historically marginalized racial and ethnic groups, and individuals disproportionately affected by adverse social drivers of health, including lower socioeconomic status, housing instability, and limited access to preventive care. Improving stroke care at SNHs presents a critical opportunity to strengthen care delivery for individuals with the greatest need. However, such efforts require a clear understanding of the barriers across all levels of the healthcare system.

In this Perspective, the authors adopt a socioecological framework to explore patient-, community-, institution-, and policy-level influences on stroke care in SNHs. Patient-level barriers include chronic disease burden, limited health literacy, and language barriers. Community-level challenges, such as neighborhood disadvantage, transportation challenges, and food insecurity, contribute to delays in care and recovery. At the institutional level SNHs often face inconsistent access to diagnostic imaging, limited specialty support, variation in stroke center certification, and staffing shortages. At the policy level, financing structures, documentation requirements, and performance metrics may unintentionally penalize under-resourced hospitals. Findings have been synthesized in this text across these domains and highlight opportunities for research, workforce development, and stroke care delivery improvement. A socioecological approach is essential to addressing disparities in stroke outcomes and guiding multilevel strategies that ensure consistent, high-quality care for underserved populations.

## Introduction

**Fig. 1.**
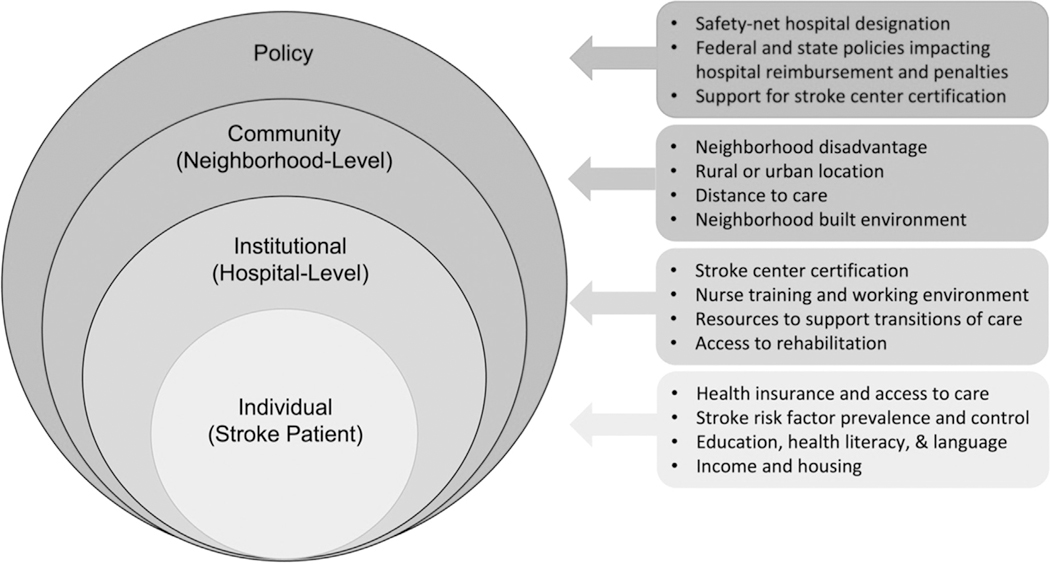
Socioecological framework for stroke care in safety-net hospItals. This framework illustrates how patient, institutional, community and policy-level factors interact to influence stroke care delivery in SNHs.

There is growing recognition of the social and behavioral factors influencing stroke outcomes. While much of the literature has focused on individual-level factors affecting access to clinical care and outcomes, less attention has been given to hospital- and health system-level influences. To address this gap, socioecological frameworks consider how factors at multiple levels, individual, institutional, community, and policy, influence behavior and disease outcomes (Fig. 1) [1]

Prior studies have largely examined patient-level or organizational factors in isolation, without fully accounting for the broader multilevel barriers in safety net hospitals (SNHs). This fragmented perspective limits our understanding of the complex challenges these institutions face.

This review explores challenges in stroke care delivery at SNHs across all levels of the socioecological model and highlights areas for future research and intervention. SNHs broadly provide care to low-income and historically disadvantaged populations in resource-limited environments [2] A 2021 systematic review by Heffner et al. identified 11 definitions of SNHs within five thematic categories (Table 1) [3,4] Medicaid Disproportionate Share Hospital (DSH) payments are statutorily required payments to hospitals from federal and state funds that offset uncompensated care costs [5] The review identifies the most commonly used definitions for SNHs as the DSH payment index (highest quartile/percent), the proportion of Medicaid and/or uninsured inpatient discharges, and nonfederal government hospital (public hospital) status. In this review, the authors adopt the broader definition while noting the specific definitions used in the referenced literature. A socioecological framework is used in this text to explore patient-, community-, institution-, and policy-level influences on stroke care in SNHs and highlight areas for future research and intervention.

## Characteristics of safety-net hospitals

SNHs differ from non-SNHs in patient demographics, hospital resources, and how they are defined. Popescu et al. found low concordance across common SNHs’ definitions (e.g., DSH status, Medicaid/uninsured caseload, uncompensated care costs), though all captured populations with greater social and clinical complexity [6] SNHs were more likely to care for historically disadvantaged racial and ethnic groups, low-income communities, and individuals receiving mental health care [6] Hospitals identified by uncompensated care had higher bad debt, charity care, and unreimbursed costs, along with lower margins [6] The DSH definition did not account for the updated 2020 Medicare formula that includes uncompensated care costs [7]

### Quality of care and stroke outcomes at safety-net hospitals

Although limited, available studies suggest disparities in some aspects of stroke care and outcomes at SNHs. One study found significantly higher 30-day readmission rates among Medicare patients hospitalized with ischemic stroke at SNHs versus non-SNHs. Participation in the Medicare Shared Savings Program (MSSP), a voluntary program that rewards organizations for delivering high-quality Medicare care, did not affect these differences [8]

Another study using Nationwide Inpatient Sample (NIS) data examined 540,558 carotid revascularization procedures, stratified by the burden on SNHs [9] High-burden hospitals had higher odds of postoperative stroke, stroke/death, non-home discharge, and increased readmission and hospitalization costs, even after adjusting for patient and hospital characteristics [9] In contrast, a 2022 CMS Care Compare analysis found no differences in 30-day mortality for stroke patients between SNHs and non-SNHs [10]

In summary, existing data point to variation in stroke treatment and outcomes at SNHs, though findings are inconsistent. Differences may reflect variability in SNHs’ characteristics, patient populations, and definitions of safety-net burden.

## Patient-level variables impacting stroke outcomes

Patients served by SNHs often have characteristics associated with worse stroke outcomes. Individuals with lower socioeconomic status (SES) and from historically disadvantaged racial and ethnic groups face a disproportionate burden of vascular risk factors and adverse social conditions that negatively impact stroke outcomes [11–13]

### Race, ethnicity, and vascular risk factors: hypertension and diabetes

Black adults in the US are more likely to have hypertension (HTN) and uncontrolled HTN compared to non-Hispanic White (hereafter referred to as White) adults [12] While Hispanic adults have lower HTN prevalence than non-Hispanic Black (hereafter referred to as Black) and White adults, the prevalence of uncontrolled HTN is similarly high [14,15] The elevated risk of incident and recurrent stroke among Black adults is partly attributable to this disparity [16] The ability to control elevated blood pressure at hospital presentation affects both eligibility for and timely access to thrombolytic therapy, resulting in lower or delayed utilization that may influence outcomes among Black patients [13,17] Diagnosed HTN and inadequate blood pressure control are also associated with systolic blood pressure variability, which adversely impacts outcomes after ischemic stroke and intracerebral hemorrhage (ICH) hospitalization [18,19]

Diabetes prevalence and control also varies by race and ethnicity, with higher prevalence in Asian, Black, Hispanic, and American Indian/Alaskan Native individuals and higher rates of control in White individuals [20,21] Elevated glucose levels at hospital presentation and diabetes prevalence are associated with worse outcomes after ischemic stroke and ICH, including higher mortality, longer hospital stays, higher readmission rates, and increased stroke recurrence [22]

While HTN and diabetes are highlighted here, disparities in other risk factors (e.g., atrial fibrillation, dyslipidemia, obesity, sleep apnea) contribute to increased stroke burden. SNHs likely manage patients with greater risk factor prevalence and poorer control, though further studies are needed to confirm these associations.

### Health literacy and language barrier

Beyond biological risk factors, patient-level social determinants, such as health literacy and language proficiency, also significantly influence stroke care and outcomes at SNHs. While adequate access to healthcare is one factor that influences stroke outcomes, limited health literacy and limited English proficiency are social factors that play a significant role in how patients with stroke and their caregivers engage and benefit from the healthcare system. Limited English proficiency (LEP) is defined as the inability “to speak, read, write, or understand the English language at a level that permits patients to interact effectively with healthcare providers ” or with the US healthcare system [23] LEP and limited health literacy are associated with disparities in access to care, use of preventive services, treatment adherence, safety events, and health outcomes [24,25]

SNHs serve many patients with limited health literacy and English proficiency, which may contribute to communication barriers that impact vascular health and other chronic diseases [24,26,27] Health literacy related to stroke includes knowledge of risk factors for stroke prevention and warning signs for stroke recognition and emergent treatment [26] Culturally tailored stroke education interventions have been developed for patients with limited health literacy and have consistently improved knowledge and recognition of stroke signs and symptoms; however, sustaining their impact over time has remained a challenge [26,28]

Research on the impact of LEP on stroke outcomes remains limited, and observational studies have not fully characterized how LEP affects healthcare access, quality of care, or outcomes. Interventions in patients’ primary languages, such as those that improve self-efficacy and stroke symptom recognition, may help reduce disparities in SNHs’ communities [29,30] Further research using validated measures of English proficiency is needed to clarify the effects of language barriers on stroke care and outcomes.

### Income and socioeconomic status

SNHs serve a higher proportion of patients with socioeconomic disadvantage [31] Individuals living in low-income communities face several obstacles in accessing medical care, which contribute to greater stroke risk and poor outcomes [32] These include the high cost of care, limited availability of appointments outside of regular working hours, and prior negative experiences with healthcare systems, such as discrimination or feeling disrespected, which may discourage patients from seeking preventive or acute care [33] Among stroke survivors, those with lower income or unemployment have higher rates of emergency department (ED) use [33] Socioeconomic barriers are also linked to higher rates of undiagnosed stroke risk factors, poorer vascular risk factor control, lower stroke literacy, and delays in seeking care.

### Housing and homelessness

Individuals experiencing homelessness and housing insecurity frequently receive care at SNHs [34] Housing insecurity and homelessness are associated with vascular risk factors and worse stroke outcomes [35] Those without stable housing often lack primary care and rely heavily on EDs [36–38] One study found that, while intravenous (IV) thrombolysis rates were similar, patients experiencing homelessness had lower use of cerebral angiography and higher mortality compared to their counterparts [39]

Multiple patient-level clinical and social variables influence stroke outcomes in SNHs. A higher burden of risk factors, compounded by health literacy challenges, income disparities, and housing insecurity, leads to delayed care and worse outcomes. These must be considered when evaluating disparities and designing interventions for patients served by SNHs.

## Community-level variables impacting stroke outcomes

SNHs often serve as the primary healthcare access point for residents of lower-income neighborhoods [6] Neighborhood-level variables, frequently measured using a neighborhood SES (nSES) index, are associated with reduced access to care and preventive services [40–42] One study found that individuals in low nSES areas were less likely to have a usual source of care or meet preventive care goals, even after adjusting for individual characteristics [43] These effects were independent of provider supply, suggesting a neighborhood-level influence beyond insurance or health literacy.

Geographic location also influences stroke risk and SNHs’ treatment. Among rural residents, stroke accounts for 7.4 % of excess mortality [44] Rural communities experience higher rates of chronic disease and limited access to emergency services contributing to delays in stroke treatment and reduced use of thrombolytic therapy or transfer for thrombectomy [45] Between 2010 and 2021, 136 rural hospitals, many of them SNHs, closed [46] These closures were driven by declining rural populations and unreimbursed care burdens, particularly in states that opted out of Medicaid expansion [47] As a result, access to acute and specialty stroke care has diminished, partly due to the inefficient geographic distribution of specialists and healthcare infrastructure [48]

In summary, communities served by SNHs have characteristics that contribute to differences in access to acute stroke care and access to health-promoting factors that are essential for primary and secondary stroke prevention. To address the needs of communities served by SNHs, strategies should go beyond individual factors and tackle community-level barriers.

Promising community-based interventions, such as mobile stroke units, community health worker programs, and neighborhood-based stroke education initiatives, have shown potential to improve stroke awareness, preparedness, and access to timely care in underserved populations [49] Additional factors such as food insecurity, the prevalence of food deserts, limited access to safe places for physical activity, and inadequate public transportation infrastructure also influence stroke risk and recovery [50] Furthermore, justice-involved individuals often face complex barriers to consistent healthcare access, which may further exacerbate disparities in SNH communities [51]

## Institution-level factors impacting stroke outcomes

SNHs play an essential role in the US healthcare system by providing healthcare to economically disadvantaged individuals. However, some SNHs may have limited economic resources to fully address the needs of the patients they serve [6] In this section, hospital characteristics that impact patient outcomes and the relationship between these factors and patient outcomes in SNHs have been discussed.

The COVID-19 pandemic further disrupted the nursing workforce, leading to a mass departure of experienced nurses and a dramatic increase in reliance on agency staffing, which raised labor costs for hospitals. SNHs, operating with smaller margins, have faced persistent challenges in attracting and retaining highly trained nurses. Additionally, the temporary reduction in clinical education requirements at some nursing programs during the pandemic may have affected staff preparedness in certain settings [52]

### Nursing staffing, training, and work environment

Multiple studies describe the critical role of nursing in stroke patient care and outcomes [24] Nurses are essential in triage, treatment, monitoring, screening, education, care coordination, and discharge. Nurse staffing, training, specialty roles, and work environment are all associated with patient outcomes. Lower nurse-to-patient ratios are linked to improved outcomes and reduced mortality [53–55] Better staffing is also associated with lower racial disparities in 30-day readmission rates after stroke [56] Inadequate registered nurse (RN) staffing is linked to reduced patient mobilization during stroke unit care, even when out-of-bed orders are present [57] Stroke-nursing certification also influences outcomes. A study comparing care by stroke-certified and non-stroke-certified RNs (SCRN) demonstrated that SCRNs were more knowledgeable, confident, and timely in activating acute stroke protocols and providing quality care [58]

Nurse staffing, leadership, and interdisciplinary relationships shape the nursing work environment, which significantly affects stroke outcomes. A national study found lower readmissions and shorter lengths of stay in hospitals with stronger nursing environments [55] These factors may be especially critical for SNHs [59] In a multi-state study, nurses serving economically disadvantaged patients reported lower unit quality, more job dissatisfaction, and higher burnout. Adjusting for the work environment eliminated the association between economic disadvantage and nurse outcomes [59] These findings underscore the importance of strengthening work environments to support care quality and staff retention in SNHs.

Due to maintenance costs and lower operating margins, SNHs may have challenges related to nursing staffing, training, and work environment [6] Despite similar nurse-to-patient ratios at SNHs and non-SNHs, one study found that SNHs saw fewer benefits from improved staffing [60] This was attributed to higher patient acuity, poorer quality in non-nursing care domains, and reliance on temporary staffing solutions like contract nurses and overtime.

Legislation is one tool that can influence hospital-level factors that affect stroke care. A study examining the impact of legislation mandating minimum nurse-to-patient ratios in California examined the differential effect on SNHs vs non-SNHs [61] SNHs had higher nurse-to-patient ratios and fewer highly trained nurses before the legislation. While compliance with mandated staffing ratios improved after legislation across all hospitals, increases in the proportion of more highly trained nurses were only observed in non-SNHs.

### Emergency department

ED characteristics can significantly influence stroke care. While the literature is mixed, EDs at some SNHs have shown higher levels of crowding, longer wait times, and lower staffing levels compared to non-SNHs, which may affect stroke diagnosis and initial treatment [62–64] ED crowding, in particular, is associated with adverse outcomes as well as all-cause inpatient mortality [65,66] Data are inconclusive regarding how these SNHs’ ED factors may affect the quality and timeliness of stroke care [67,68] Multidisciplinary interventions show promise for overcoming stroke treatment delays that may be seen in SNHs’ EDs [69]

### Stroke coordinators

Stroke coordinators, who are often, but not always nurses, work in various clinical areas in hospitals and play essential roles in the care of stroke patients. They are often responsible for facilitating patient care from the time of presentation to the time of discharge and beyond. Stroke coordinators play critical roles in maintaining Stroke Center certification, data tracking for adherence to quality metrics, development of stroke pathways and policy, discharge coordination, and patient and nursing education [70] While data on the impact of stroke coordinators on patient outcomes are lacking, one study showed that stroke patients receiving care at hospitals with a stroke coordinator were more likely to have education on risk factor modification, early assessments for rehabilitation, and a discharge care plan [71] National and international guidelines endorse and outline a stroke coordinator’s role in a stroke center; however, the role comes with additional costs, which may make this role difficult for certain SNHs to support [72]

### Radiology

SNHs face growing fiscal constraints that may impact radiology services [73] Imaging equipment and technology often vary in age, sophistication, and capability [73–77] Most CT scanners are for general-purpose imaging of all anatomic regions, which makes them more common in EDs due to their reliability and minimal downtime. Limited data exist on the availability of advanced neuroimaging for stroke patients at SNHs; however, known challenges such as outdated staffing models and CT technologist shortages may further impact imaging capabilities at SNHs [74,75] More research is needed to understand how imaging resources impact stroke care and outcomes at SNHs.

### Stroke center certification

Stroke Center certification is a hospital-level variable associated with improved quality metrics and patient outcomes [78,79] There are currently five different stroke center designations that hospitals can achieve: acute stroke-ready hospital (ASRH); primary stroke center (PSC); Primary Plus Stroke Center (PSC +); Thrombectomy-capable stroke center (TSC); and Comprehensive Stroke Center (CSC) [80,81] Data show that hospitals with stroke center designation have shorter time from door-to-first imaging, higher rates of appropriate IV thrombolysis, and shorter door-to-needle times compared to data from before designation. Stroke Center designation is also associated with improved in-hospital mortality, lower 30-day mortality, and lower 30-day readmission rates compared to non-certified hospitals [82,83]

Evidence from the Field Administration of Stroke Therapy-Magnesium (FAST-MAG) showed that hospitals only saw improvements in stroke metrics after certification, suggesting that the certification process, not just pre-existing characteristics, drives care improvements [84]

Given the demonstrated benefits of stroke center certification on patient outcomes, certification is one hospital factor that may improve outcomes for patients served by SNHs. There are gaps in knowledge regarding the proportion of SNHs that are certified stroke centers; however, available data suggest that populations served by SNHs have lower access to hospitals with stroke center certification. One study found that hospitals in low-income service areas and those with a lower percentage of profit margin distribution were less likely to adopt stroke certification [85] Further, a recent study showed that hospitals in socioeconomically disadvantaged communities were less likely to obtain stroke certification [86] Several studies have shown that rural hospitals are less likely to be stroke-certified compared to urban hospitals, and patients in rural areas live farther from certified stroke centers [80,87,88]

Economic factors may present barriers to adopting and maintaining stroke center certification. Certification costs include those necessary for certification (payments to certifying bodies), infrastructure, personnel costs, and development and maintenance of data registries. Stroke center designation can also influence EMS logistics, which may lead to increased stroke patient volume at the destination hospital. Consideration of the infrastructure needed to manage complex stroke patients may influence decisions around stroke certification [89]

### Processes to support transitions of care and post-stroke care

In addition to acute stroke and in-hospital care, SNHs face barriers in providing effective transitions of care from hospitalization to outpatient settings, which are essential for reducing morbidity and preventable readmissions after stroke. In a national sample of US hospitals, metrics related to care transitions were poor at SNHs compared to non-SNHs. Patients at SNHs reported more gaps in discharge information and poorer communication with physicians, and SNHs also had lower odds of meeting value-based performance benchmarks [87]

Hospital readmission is a particular challenge for SNHs [88] A survey of hospital leaders identified differences in the care processes that influence readmission. SNHs were less likely to have internal tracking systems for reducing readmissions and less likely to track hospital readmissions based on race and ethnicity [88] Among SNHs, higher-performing hospitals were more likely to use electronic tools for discharge medication reconciliation and discharge coordinators to ensure care continuity. This study suggests that, despite a higher burden of patient-related factors contributing to poor care transitions and higher readmission rates, these issues can be mitigated by processes known to improve care transitions [88]

Post-stroke care is critical for secondary stroke prevention, yet uninsured and underinsured patients treated at SNHs often face barriers to primary and specialty follow-up. These challenges contribute to higher readmission rates and worse long-term outcomes [90] More research is needed on how SNHs facilitate post-stroke care and whether disparities in access affect secondary prevention.

### Access to rehabilitation

National guidelines recommend intensive stroke rehabilitation to improve functional outcomes and quality of life [91] Inpatient and outpatient rehabilitation services are under-utilized in stroke patients for numerous reasons, including therapy availability, insurance status, and geographic variability [92] Stroke patients who are managed at SNHs may face additional barriers to rehabilitation access. Uninsured stroke patients have less access to institution-based rehabilitation services (skilled nursing facility or inpatient rehabilitation facility) than stroke patients with private insurance [93–95] Such patients often have no access unless the hospital pays, provides home health, or sends the patient to a Medicaid-pending facility or home with family training. While stroke patients with Medicaid insurance have better access to institution-based services compared to the uninsured, they have less access to more intensive inpatient rehabilitation than those with private insurance [96] Unless SNHs have an associated inpatient rehabilitation facility for patients with Medicaid or uninsured, these hospitals may have more patients discharged from acute care with less-than-optimal rehabilitation services. More studies are needed to understand access to institutional and outpatient rehabilitation services for patients discharged from SNHs.

In summary, SNHs face several institution-level barriers affecting stroke care and outcomes. These include staffing challenges (e.g., nursing and CT technicians), suboptimal nursing environments, ED crowding and wait times, limitations in imaging technology availability and maintenance, and economic hurdles in achieving and maintaining stroke certification. Additionally, SNHs struggle with coordinating post-stroke care, as uninsured and underinsured patients often have limited access to rehabilitation services, resulting in higher readmission rates and poorer long-term recovery. Practical strategies to improve stroke care at SNHs must address these challenges.

## Policy-level considerations for safety-net hospitals

There are extensive gaps in knowledge related to the influence of local, state, and national policies on stroke outcomes at SNHs. One factor that limits the ability of researchers to examine the influence of health policy is the lack of a standard definition for SNHs. Because external patient and community factors influence hospital performance, some researchers propose consideration of various markers of social disadvantage when determining a comprehensive definition of SNHs [97] Because hospital reimbursement and penalties are influenced by how SNHs are defined, failure to capture critical social factors that adversely influence health outcomes in SNHs’ characterization may lead to lower reimbursement and/or higher penalties for hospitals serving patients and communities with substantial social needs [97] Failure to account for social risk can lead to disproportionately higher penalties for safety-net hospitals [98] Consideration of social risk factors and proportionate compensation to hospitals can lead to equal or greater benefits for patients served at SNHs.

Several federal programs can potentially influence stroke care and outcomes at SNHs. These programs include the Hospital Readmissions Reduction Program, the Hospital-Acquired Conditions Reduction Program, the Hospital Value-based Purchasing Program, and the Medicare Shared Savings Program (Table 2) [8,98,99] Federal value-based payment models may inadvertently penalize SNHs by failing to adjust for social risk, reinforcing resource gaps [99]

Health policy at the state level plays a vital role in stroke care. Research has shown that state stroke legislation is associated with stroke center certification. Uchino et al. reported that 18 states with specific legislation related to stroke center designation and stroke triage regulation had a larger proportion of PSCs than states without legislation [100] Methods of certification across states vary, with some states only recognizing stroke centers certified by nationally certifying bodies and some by state certification programs [80] State and federal initiatives to support SNHs in maintaining stroke center certification is another example of a policy-level initiative with the potential to impact stroke care and outcomes at SNHs; however, more research is needed in this area.

The Centers for Disease Control and Prevention (CDC) performed an impact analysis of stroke systems of care (SSOC) state policy interventions to improve access to time-sensitive stroke treatment. States with at least one SSOC policy in effect demonstrated better performance than expected in the absence of any SSOC policy on these stroke metrics: higher proportion of certified PSCs, higher brain imaging rates within 45 min of presentation, lower in-hospital costs, and lower in-hospital mortality [101,102] The report outlines specific policy interventions that predicted better stroke outcomes, such as requirements for an SSOC task force and a statewide continuous quality improvement data and reporting system. While the analysis was not specific to SNHs, this information can be used to develop baseline expectations for stroke care across the US and inform state-specific goals and process implementation to achieve the best outcomes for stroke patients.

Emerging threats to Medicaid funding at the state and federal levels pose additional risks to safety-net hospitals. Reductions in Medicaid support could result in an expanded uninsured population, placing further financial strain on hospitals that already operate with limited margins [103] These shifts may exacerbate disparities in access and continuity of care in SNHs’ communities.

In summary, state and federal policies can improve stroke care at SNHs. The absence of a standard definition for SNHs complicates research in this area. Policies must account for social and community factors that influence stroke outcomes, independent of hospital factors. Federal quality programs may disproportionately penalize SNHs, while participation barriers can limit benefits. State-level policies can help to standardize stroke care and improve outcomes, but implementation and monitoring barriers, as well as the specific needs of SNHs, must be addressed.

## Research gaps and future directions

**Fig. 2.**
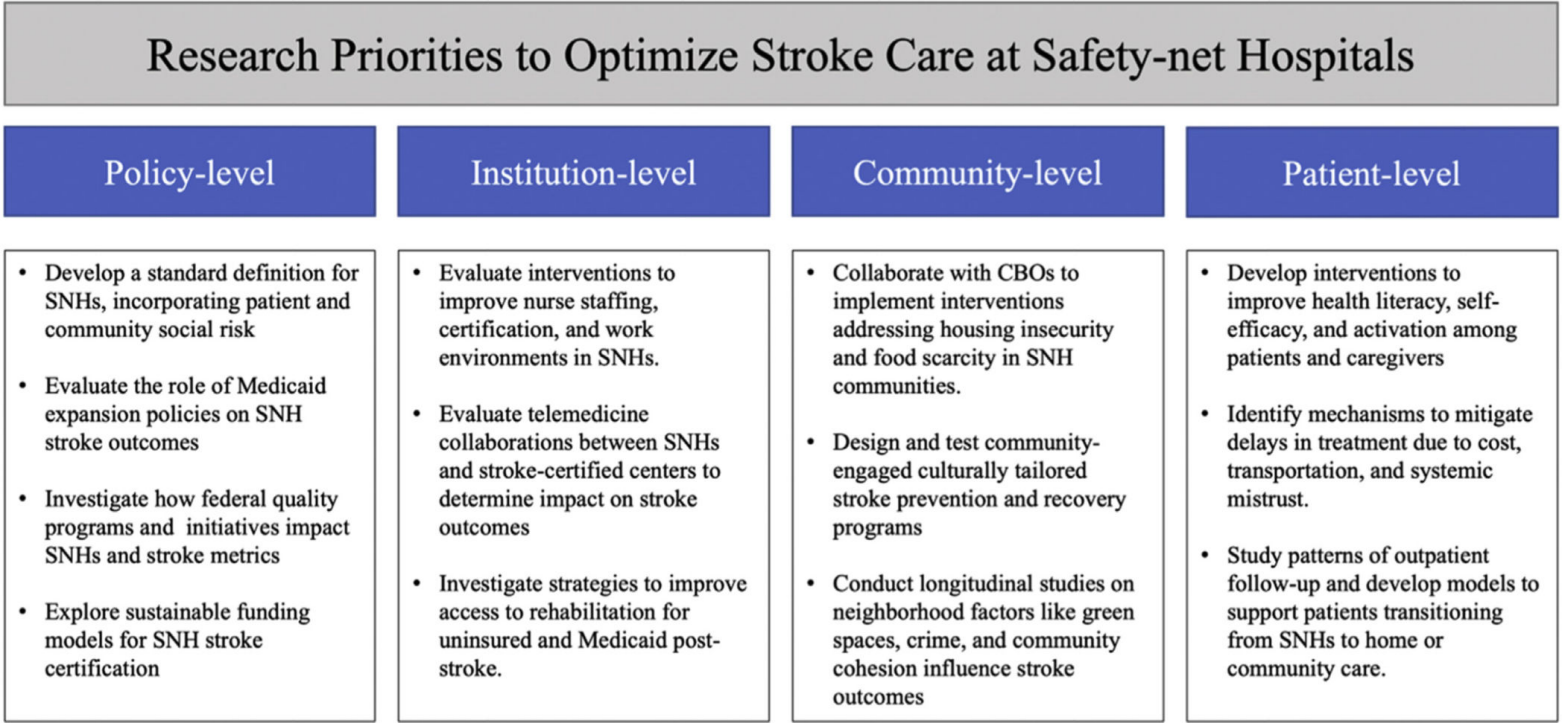
Future research priorities in stroke care at safety-net hospitals. Key areas for investigation across multiple levels of the socioecological model, based on observed gaps in patient outcomes, systems delivery, and policy infrastructure.

While a substantial review of data regarding the multilevel barriers to care at SNHs using a socioecological framework is provided here, more research using rigorous methods with various approaches is needed to fully characterize patient, community/geographic, hospital/health system, and policy level factors and their influence on stroke care and outcomes at SNHs. To this end, the authors suggest several future areas of study (Fig. 2). While these suggestions are not exhaustive, they serve as a starting point for generating new knowledge regarding stroke care and outcomes at SNHs.

Despite a growing body of research on stroke disparities, several gaps remain. First, there is a lack of longitudinal studies evaluating the effectiveness of SNH-specific stroke interventions. Second, SNHs remain underrepresented in multicenter stroke trials, limiting the generalizability of findings to high-need populations. Third, hospital-level process variation —such as differences in staffing, infrastructure, or care coordination —remains poorly understood and may contribute to outcome disparities across SNHs.

Addressing stroke disparities in SNHs requires coordinated, multilevel strategies. As illustrated in the socioecological framework (Table 3), actions are needed across policy, institutional, community, and individual levels. Policy and institutional strategies include incentivizing stroke center certification, adopting equity-centered practices, tracking disparities, and building multidisciplinary teams [104] Community partnerships should address food insecurity, housing, transportation, and language barriers. Educational efforts that enhance self-efficacy and leverage social networks are also essential. Finally, health services and community-engaged research are critical to evaluating and scaling models for equitable stroke care delivery.

## Conclusions

The patients served at SNHs are from the same populations in whom disparate stroke outcomes across the stroke care continuum are pervasive. SNHs are critically important in providing stroke care to patients with a disproportionate burden of adverse social drivers of health who are also at the highest risk for stroke. An enhanced focus on the challenges of stroke care at SNHs provides the opportunity to develop interventions at multiple levels to improve stroke outcomes in these groups, thereby contributing to national efforts to eliminate stroke disparities and the societal and individual burden of this disease [105] Targeted investments in SNHs and multilevel strategies to strengthen stroke care delivery represent a critical opportunity to reduce national disparities, advance health justice, and drive innovation in equitable stroke systems of care.

## Figures and Tables

**Table 1:** Summary of common operational definitions for safety-net hospitals.

Operational Definitions	Thematic Categories
DSH Payment index (top quartile/top 20 %)	DSH payments
Medicaid and Uninsured Inpatient Discharges (top quartile)	Medicaid caseload, Uncompensated care
Medicaid Inpatient Discharges (one standard deviation above the state median/mean)	Medicaid caseload
Medicaid Inpatient Discharges (one standard deviation above state mean) OR Nonfederal Government Hospitals (Public Hospitals)	Medicaid caseload, facility characteristics
AHRQ SES Index	Patient case mix
The ratio of bad debt plus charity care (top decile) OR uncompensated care expenses	Uncompensated care
Teaching COTH Member OR Nonfederal Government (Public Hospitals)	Facility characteristics
Medicaid Inpatient Discharges (top quartile) OR DSH status (y/n) OR proportion of charges for charity care	Medicaid caseload, Uncompensated care, DSH payments
Percentage of patients eligible for Supplemental Security Income (SSI)	Patient case mix
Nonfederal Government Hospitals (Public Hospitals)	Facility characteristics
Medicare uncompensated care burden (top quartile)	Uncompensated case

Abbreviations: DSH, Disproportionate Share Hospital; COTH, Council of Teaching Hospitals and Health Systems; AHRQ SES index, Agency for Healthcare Research and Quality’s Socioeconomic Status Index.

* Adapted from Hefner et al.^3^.

**Table 2:** Overview of medicare value-based purchasing programs relevant to stroke care at safety-net hospitals[8,98,99].

Program	Summary
Hospital Readmissions Reduction Program	• Financial penalties are given to hospitals with higher-than-expected readmission rates for select conditions: acute myocardial infarction, heart failure, pneumonia, chronic obstructive pulmonary disease, total knee or hip arthroplasty, and coronary artery bypass grafting • Penalties applied to Medicare base-operating DRG payments
Hospital Value-Based Purchasing Program	• Annual withholding of a portion of Medicare payments from participating hospitals for inpatient services • Redistribute withholdings to fund value-based incentive payments to participating hospitals based on quality measure performance
Hospital-Acquired Condition Reduction Program	• Hospitals are penalized 1 % of their total inpatient Medicare revenues to reduce the incidence of infections and other adverse safety events.
Medicare Shared Savings Program	• Accountable care organizations can opt into a voluntary program that provides financial incentives to increase coordination of care and reduce unnecessary costs for Medicare fee-for-service beneficiaries

∗ The programs identified here impact the quality of inpatient care and have the potential to impact stroke care (indirectly or directly).

DRG: Diagnosis-Related Group.

**Table 3:** Proposed multilevel strategies to improve stroke care in safety-net hospitals.

Policy	Establish resources to support SNHs in establishing and maintaining stroke center certification Establish CMS equity outcome measures to incentivize institutions that address health equity in the context of stroke care. Support improvement in SNH infrastructure and data tracking processes. Implement policies to enforce language accessibility tracking. Engage the pharmaceutical industry to prioritize impactful and equitable work in healthcare and allocate funding accordingly. Inform congressional representatives about impactful and equitable healthcare initiatives to encourage funding that promotes health equity. Create policy incentives for clinical research collaboration with SNHs to reduce the exclusion of SNH patients from clinical trials.
Community	Collaborate with community-based organizations to identify community needs (educational/access/resource/etc.) in the context of stroke preparedness, prevention care, and survivorship. Partner with community-based organizations to enhance primary and secondary stroke prevention efforts in SNHs such as healthy food stores and access to recreational spaces Equip community members and leaders with relevant knowledge about emergent stroke care in ways that align with their needs. Support community organizations that address non-medical drivers of health (food insecurity, housing insecurity, transportation needs, etc.). Enhance access to rehabilitation centers, gyms, YMCAs, and other organizations that may support stroke recovery needs
Institutional	Pursue opportunities to achieve resources for stroke center certification based on patient and community needs and hospital resources. Create telemedicine collaborations with stroke-certified hospitals or centers to extend expert care to underserved areas. Foster team-based science and interdisciplinary stroke training programs to improve consistency and quality of care across hospitals, regardless of certification designation. Allocate resources to build institutional research capacity and enable participation in clinical studies, which can support infrastructure for future research initiatives. Form equitable collaborations with academic institutions aiming to conduct clinical research to ensure mutual benefit and representation. Implement ready-to-use, non-English language print materials and adopt hospital policies to track language usage and accessibility data effectively. Provide targeted stroke training for nurses, focusing on acute care protocols, stroke recognition, and post-stroke recovery management. Utilize community health workers as liaisons between hospitals and communities to improve stroke prevention efforts and enhance education on stroke care.

Abbreviations: SNH, Safety-net hospital; CMS, Centers for Medicare and Medicaid Services; SCC, Stroke Certification Center.
